# A case report of Fontan procedure-related hepatocellular carcinoma: pure laparoscopic approach by low and stable pneumoperitoneum

**DOI:** 10.1186/s12893-020-00741-8

**Published:** 2020-04-21

**Authors:** Yuki Yokota, Takehiro Noda, Shogo Kobayashi, Kenichi Matsumoto, Yoshihiro Sakano, Yoshifumi Iwagami, Yoshito Tomimaru, Hirofumi Akita, Kunihito Gotoh, Koji Umeshita, Yuichiro Doki, Hidetoshi Eguchi

**Affiliations:** 1 Department of Gastroenterological Surgery, Graduate School of Medicine, Osaka University, 2-2, Yamadaoka, Suita, Osaka, 565-0871 Japan; 2 Division of Health Science, Graduate School of Medicine, Osaka University, Osaka, Japan

**Keywords:** Fontan procedure, Hepatocellular carcinoma, Laparoscopic hepatectomy

## Abstract

**Background:**

The Fontan procedure has become the standard operation for patients with single ventricle physiology. Due to cardiac hypokinesis and high central venous pressure, laparoscopic approach, especially in hepatectomy, was considered as controversial after the Fontan procedure. We presented a case of hepatocellular carcinoma (HCC) that was successfully treated by pure laparoscopic hepatectomy with stable pneumoperitoneum after the Fontan procedure.

**Case presentation:**

An 18-year-old man was referred to our hospital for examination of a hepatic tumor. The patient underwent the Fontan procedure for single ventricle physiology at 6 years of age. Abdominal contrast-enhanced computed tomography (CT) revealed a hypovascular mass in segment 2 and a hypervascular mass in segment 4 of the arterial phase, followed by a delayed washout. CT arteriography revealed that both masses showed hypervascular tumors, and CT during arterial portography showed that both were low-density masses. The patient’s general condition was good, and cardiac and respiratory functions were well maintained. Pure laparoscopic hepatectomy was safely performed by keeping the pneumoteritoneum pressure under 6–8 mmHg and monitoring central venous pressure (11–21 mmHg) and end-tidal carbon dioxide. The Pringle maneuver was applied during hepatic resection. The non-anatomical resections were completed without intraoperative complications. The patient was discharged on the 9th postoperative day without postoperative complications.

**Conclusions:**

Our report suggests that treatment of HCC by pure laparoscopic hepatectomy after Fontan circulation can be safely performed in patients under sufficient circulatory management.

## Background

The Fontan procedure has become the standard operation for patients with single ventricle physiology. Fontan-associated liver disease, such as hepatic fibrosis, cirrhosis or hepatocellular carcinoma (HCC), is one of the late complications in patients after the Fontan procedure [1, 2]. There have been increasing reports of HCC developing in the background of hepatic congestion and liver cirrhosis after the Fontan procedure [3–6]. Laparoscopic surgery in patients with Fontan circulation is a hemodynamic challenge because the venous return may be compromised by insufflation of carbon dioxide into the abdomen, use of the reverse Trendelenburg position, and positive pressure ventilation. Recently, there have been reports about laparoscopic surgeries such as cholecystectomy, pheochromocytoma excision and Morgani hernia after the Fontan procedure [7–9]. However, there was a potential risk by difficulty of bleeding control because of high central venous pressure (CVP) in addition to congestive liver and liver cirrhosis, and laparoscopic hepatectomy after the Fontan procedure have rarely been reported [6].

Here, we report a successfully treated case of HCC after the Fontan procedure by pure laparoscopic hepatectomy with low and stable pneumoperitoneum.

## Case presentation

The patient was an 18-year-old male. He was diagnosed with single ventricle asplenia and left inferior vena cava before birth, and underwent the Fontan procedure for single ventricle physiology at 6 years of age. He was regularly checked up after the Fontan procedure. At 18 years old, a hepatic tumor was detected by ultrasound sonography. He was referred to our hospital for treatment of the hepatic tumor.

On laboratory evaluations, the blood test results were as follows: platelet count of 22.6 × 10^4^/μL, prothrombin time of 73%, serum albumin level of 4.8 g/dL, aspartate transaminase level of 32 IU/L, alanine transaminase level of 33 IU/L, and total bilirubin level of 0.6 mg/dL. The tumor markers were alpha-fetoprotein of 3 ng/mL and des-γ-carboxy prothrombin level of 41 mAU/mL. Both hepatitis B virus antigen and hepatitis C virus antibody were negative. The indocyanine green retention rate at 15 min was 14%. The liver function was preserved, and Child-Pugh classification was categorized as A.

**Fig. 1:**
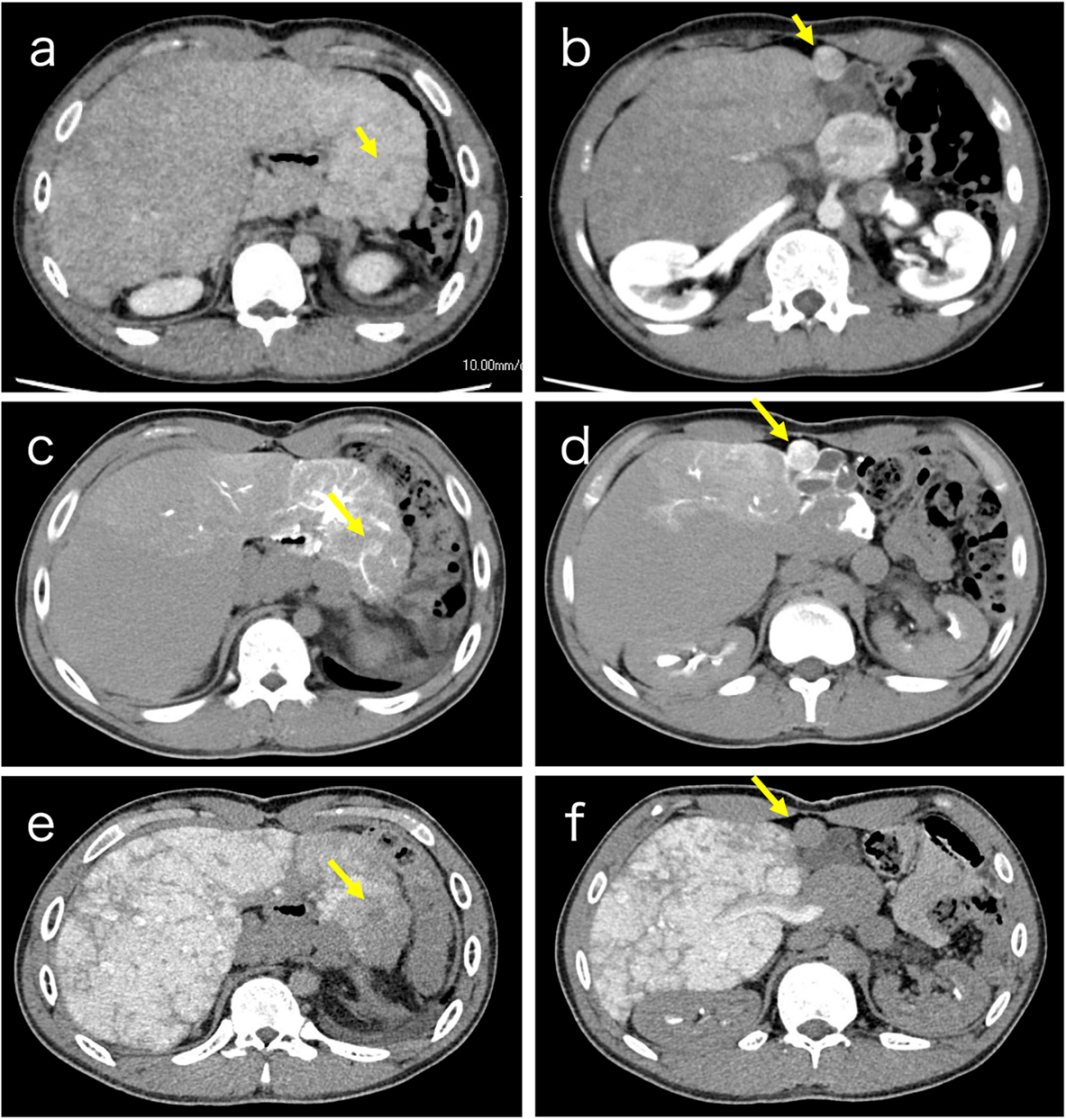
Abdominal contrast-enhanced CT and CT arteriography and arterial portography. Abdominal contrast-enhanced CT examination revealed a hypovascular mass in segment 2 (**a**), and a hypervascular mass in segment 4 on arterial phase, followed by delayed washout (**b**). CT arteriography showed that both masses were hypervascular tumors (**c**: segment 2, **d**: segment 4), and CT during arterial portography showed that both were low-density masses (**e**: segment 2, **f**: segment 4)

Abdominal contrast-enhanced computed tomography (CT) examination revealed a hypovascular mass in segment 2 and a hypervascular mass in segment 4 of the arterial phase, followed by a delayed washout (Fig. 1a, b). Gadolinium ethoxybenzyl diethylenetriamine pentaacetic acid-enhanced magnetic resonance imaging showed similar findings. CT arteriography revealed that both masses were hypervascular tumors (Fig. 1c, d), and CT during arterial portography showed that they were both low-density masses (Fig. 1e, f). Both tumors were suspected to be HCC, and thus we planned to perform hepatectomy.

Oxygen saturation in room air was 92%, and non-invasive blood pressure was 98/60 mmHg. Preoperative angiography revealed CVP of 20 mmHg and a hepatic wedge pressure of 23 mmHg. Left ventricular ejection function was estimated as 57%. Cardiac output was 3.5 L/min. After multidisciplinary medical staff meetings by adult congenital cardiologists, hepatobiliary surgeons, and anesthesiologists, the patient could be tolerated for general anesthesia, and we planned laparoscopic hepatectomy.

**Fig. 2:**
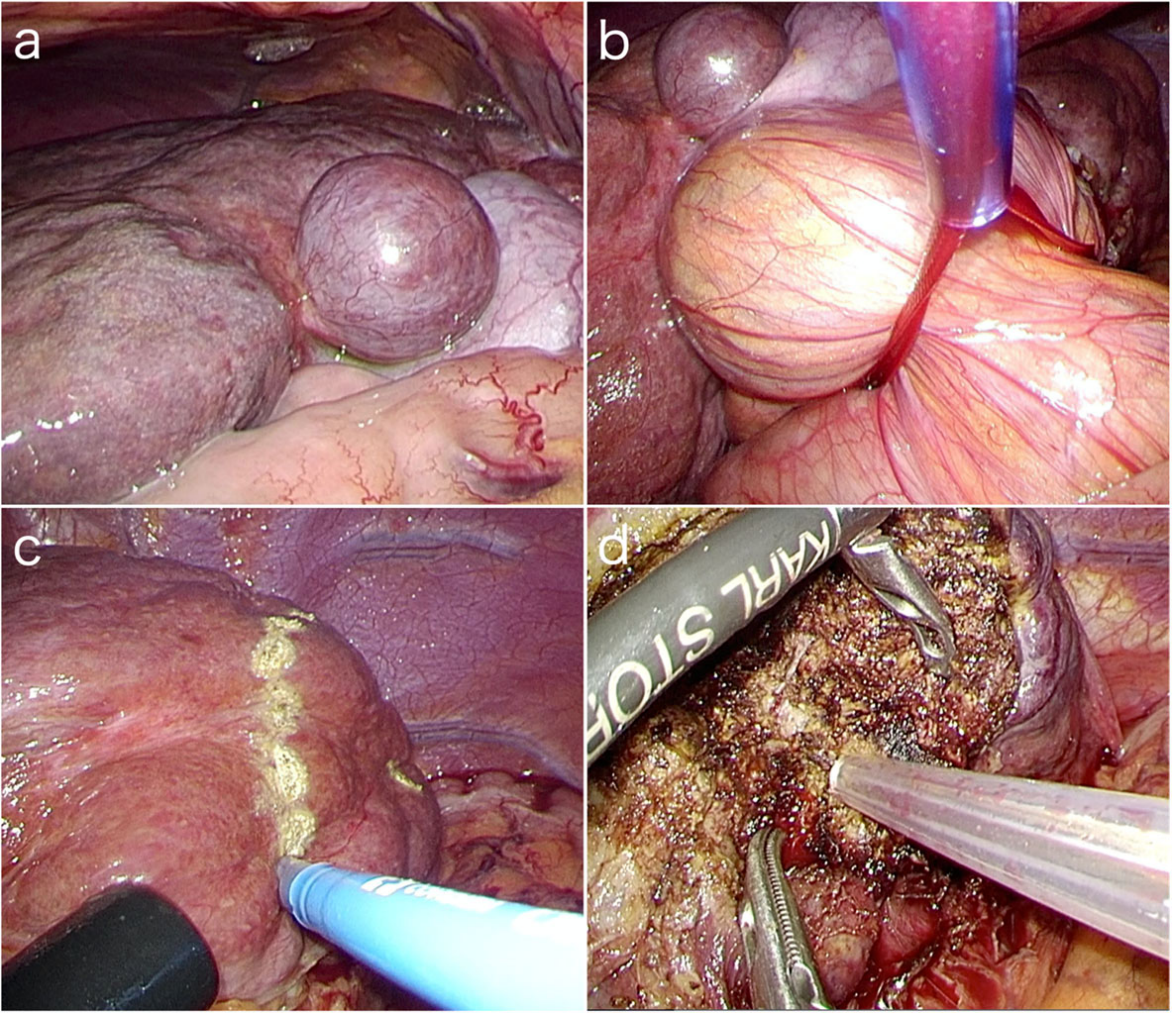
Intraoperative findings. The liver revealed hepatic congestion due to Fontan circulation (**a**). The Pringle maneuver was applied during hepatic resection (**b**). The laparoscopic non-anatomical resections were performed(**c**, **d**)

**Fig. 3:**
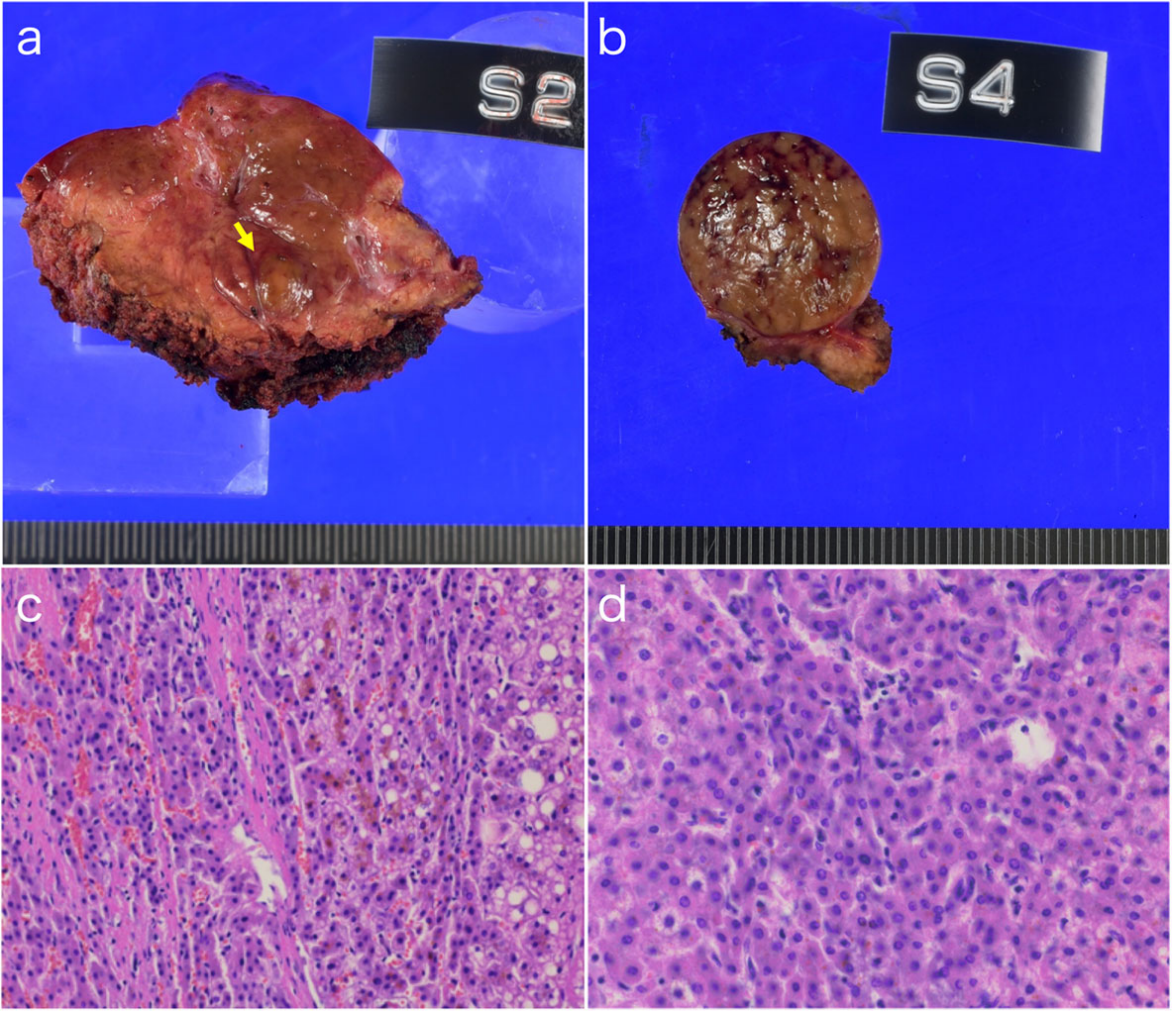
Macroscopic and microscopic findings of the resected tissue specimen. Round-shaped brown tumor in segment 2 (**a**) and segment 4 (**b**). Pathological examination revealed that the segment 2 lesion was a high-grade dysplastic nodule (**c**) and the segment 4 lesion was well-differentiated HCC (**d**)

We performed pure laparoscopic hepatectomy. Pneumoperitoneum was performed with insufflation management system (AirSeal iFs; Conmed Corp., Utica, NY, USA) for low and stable pressure was started from 6 mmHg and controlled up to 8 mmHg. The intraoperative CVPs shifted 11–21 mmHg and exceeded the pneumoperitoneum pressure. The end-tidal carbon dioxide tension shifted 36–40 mmHg. The liver revealed hepatic congestion due to Fontan circulation (Fig. 2a). The two 12 and two 5 mm ports were placed in the right and left paramedian and right and left subcostal positions. The Pringle maneuver was applied during hepatic resection (Fig. 2b). The resection line was marked, and hepatic transection was performed by laparosonic coagulating shears (Ethicon Endo-Surgery, Cincinnati, OH, USA), an ultrasonic surgical aspirator (CUSA; Cavitron Lasersonic Corp., Stamford, CT, USA), and a bipolar clamp coagulation system (VIO 300D; ERBE Elektromedizin, Tübingen, Germany) (Fig. 3c, d). The laparoscopic non-anatomical resections were completed. Then the tumors were extracted using a plastic bag through the umbilical trocar incision. A drainage tube was placed for the liver-resected surface. The operative and pneumoperitoneum times were 327 and 263 min, respectively. The intraoperative blood loss was 20 mL.

The patient was discharged on the 9th postoperative day without postoperative complications. The resected specimens showed round-shaped brown tumors and the pathological findings were revealed that the segment 2 lesion was high-grade dysplastic nodule and the segment 4 lesion was well-differentiated HCC. The patient remains free of disease at the 1-year follow-up.

## Discussion and conclusions

The Fontan operation was first described by Dr. Fontan in 1971 [1], and has become the standard surgical procedure for patients with single ventricle defects. Circulation after the Fontan procedure has the following features: nonpulsatile pulmonary perfusion, systemic venous hypertension, and low cardiac output. However, several late complications after the Fontan procedure have been observed. HCC is one of the late complications in patients after the Fontan procedure, and develop as a result of increased hepatic venous pressure, tissue hypoxia, and decreased cardiac output [2].

There are several reports of hepatectomy for HCC after Fontan procedure. The majority of them were open laparotomy procedures [3–5], and only one laparoscopic liver resection is reported for HCC on the liver surface [6]. We safely completed laparoscopic hepatectomy for HCC in the deep liver parenchyma with low and stable pneumoperitoneum pressure by using insufflation management system. Laparoscopic hepatectomy is widespread worldwide as it is less invasive and leads to early recovery and less pain after the operation. But for Fontan circulation, laparoscopic surgery is generally considered to be disadvantageous. Venous return is inhibited by increases in intra-abdominal pressure in the pneumoperitoneum due to carbon dioxide, increases in intrathoracic pressure due to diaphragm elevation, and increases in intrathoracic pressure by positive pressure ventilation. Moreover, absorption of carbon dioxide from the peritoneum increases blood carbon dioxide partial pressure and increases pulmonary vascular resistance. Carbon dioxide in the abdominal cavity might be sucked into the injured hepatic veins, causing pulmonary embolism. In this way, laparoscopic surgery is considered disadvantageous for Fontan circulation. To avoid these situations, several reports have recommended keeping the pneumoperitoneum pressure below the CVP (< 10 mmHg), managing fluid administration to maintain the low CVP, and providing appropriate ventilation so that intrathoracic pressure is not increased. In this case, we took care to keep the pneumoperitoneum pressure steady, not too high. We carefully monitored the intraoperative CVP and kept the pneumoperioneum pressure under CVP in all the time of operation. The circulatory dynamics parameters were checked in the several situation such as the change of body position, starting the pneumoperioneum, the vascular clamping and unclamping of hepatic hilum. For safety operations, it is necessary to have adequate preoperative assessments of the Fontan circulation and cooperation with other medical staff.

In conclusion, our report suggests that laparoscopic hepatectomy for HCC patients with Fontan circulation can be safely performed in selected patients with sufficient cardiac reserve. A long time has passed since Fontan surgery was first performed, and the cases of HCC with congested liver cirrhosis is expected to increase in the future. Moreover, the recurrence of HCC might be inevitable due to congested liver cirrhosis and the carcinogenesis in younger age. The minimally invasive surgery of laparoscopic hepatectomy is desirable for repeat surgery and keeping the quality of the life after surgery. The accumulation of case reports of hepatectomy for HCC with Fontan circulation is needed to investigate the long-term prognosis.

## Data Availability

The data supporting the findings of this study are available within the article.

## Abbreviations

HCC: Hepatocellular carcinoma
CT: Computed tomography
MRI: Magnetic resonance imaging
CVP: Central venous pressure
EtCO_2_: End-tidal carbon dioxide

## References

[1] Fontan F, Baudet E (1971). Surgical repaire of tricuspid atresia. Thorax.

[2] Gnanappa GK, Celermajer DS, Sholler GF (2017). The long-term Management of Children and Adults with a Fontan circulation: a systematic review and survey of current practice in Australia and New Zealand. Pediatr Cardiol.

[3] Oh C, Youn JK, Han JW (2016). Hepatocellular carcinoma after the Fontan procedure in a 16-year-old girl: a case report. Medicine (Baltimore).

[4] Takuma Y, Fukada Y, Iwadou S (2016). Surgical resection for hepatocellular carcinoma with cardiac cirrhosis after the Fontan procedure. Intern Med.

[5] Lo KS, Chan MY, Ma KW (2018). Left hepatectomy in a patient with a Fontan circulation. Transl Gastroenterol Hepatol.

[6] Angelico R, Lisignoli V, Monti L (2019). Laparoscopic liver resection for hepatocellular carcinoma in Fontan-associated chronic liver disease. The first case report. Int J Surg Case Rep.

[7] McClain CD, McGowan FX, Kovatsis PG (2006). Laparoscopic surgery in a patient with Fontan physiology. Anesth Analg.

[8] Lee HC, Nam K, Lee JH (2014). Anesthetic management of laparoscopic pheochromocytoma excision in a patient with a Fontan circulation: a case report. Korean J Anesthesiol.

[9] Halaweish I, Ralls M, Siddiqui S (2015). Obstructive jaundice secondary to Morgagni hernia in an infant with Fontan circulation. Pediatr Surg Int.

